# Use of Ultrasound in Reconditioning by Welding of Tools Used in the Process of Regenerating Rubber

**DOI:** 10.3390/ma11020276

**Published:** 2018-02-10

**Authors:** Dan Dobrotă, Valentin Petrescu

**Affiliations:** Faculty of Engineering, Lucian Blaga University of Sibiu, Sibiu 550024, Romania; valentin.petrescu@ulbsibiu.ro

**Keywords:** process, reconditioning, welding, ultrasounds, tools, regenerated rubber

## Abstract

Addressing the problem of reconditioning large parts is of particular importance, due to their value and to the fact that the technologies for their reconditioning are very complex. The tools used to refine regenerated rubber which measure 660 mm in diameter and 2130 mm in length suffer from a rather fast dimensional wear. Within this research, the authors looked for a welding reconditioning procedure that would allow a very good adhesion between the deposited material layer and the base material. In this regard, the MAG (Metal Active Gas) welding process was used, but the ultrasonic activation of the welding process was also considered. Thus, the wire used for welding was activated considering a variation of the frequency of ultrasounds in the range f = 18–22 kHz respectively of the oscillation amplitude *A* = 30–60 μm. Under these conditions it was found that the presence of ultrasonic waves during the welding cladding process results in uniform deposition of hard carbons at the grain boundary and in the elimination of any existing oxides on the deposition surface, but at the same time increases the adhesion between the base material and the additional material, all of which positively influence the wear and corrosion resistance of the tools used to refine the regenerated rubber.

## 1. Introduction

The processes for superior utilization of rubber waste are complex processes that use specialized construction equipment and tools. Thus, an important operation of the technological process of transforming rubber waste into regenerated rubber is represented by the refining process. The tools needed for the equipment used in the rubber refining process are of a cylinder form and subject to very high mechanical stresses. Thus, the wear on the tools used in refining the regenerated rubber is mainly due to the dry rolling phenomena occurring between a metal and a non-metal.

The wear that appears on these type of tools is unidimensional and is characterized by the fact that during the rubber refining processes a part of the tool material mass is lost which causes a reduction in its size. This dimensional wear of the tools can be compensated by adjusting the distance between them, but the tool size reduction in the radial direction cannot exceed 20 mm. The prevailing influence on dimensional wear is the efforts that accompany the process of refining rubber waste. Due to the fact that the cylindrical tools used for refining rubber waste are quite large (diameter 660 mm and length 2130 mm), and are executed from special steel materials, it has always been an issue finding the technologies for their refurbishment [1].

There are situations when the regular dimensional wear process may be replaced by a very aggressive one, but only for a short period of time in the case that an abrasive material is present between the moving parts (metal inserts, hard bodies from various materials). The characteristics of the tool material and of the refined material have a strong influence on the size of the dimensional wear. Proper choice of chemical composition, alloying and heat treatment for the tool material and the reconditioning process can have a favorable influence on the increase in wear resistance [2].

A possible technology to be used for reconditioning these tools is welding cladding, and this is due to the fact that the refurbishment must be done by depositing a layer of material with a pretty high thickness. Thus, in order to be able to apply in optimum technical and economic conditions a specific process of refolding by welding the parts used in different industrial branches, the reconditioned part has to have a lifetime and a reliability close to or even better than the new piece. Obtaining a technically refurbished piece is possible if the deposited material creates, together with the base material, a system that has a similar operating behavior to that of a new piece [3,4].

The process of ultrasonic welding refurbishment of complex parts of relatively large dimensions determines an increase in the resistance and reliability of used parts subject to the reconditioning process. This can happen if the deposited material creates, together with the base material, a partner system with a proper behavior to the operating demands of the reconditioned part [5].

It should also be taken into account that the parameters of the welding of the technological loading process must be appropriately established in order to obtain a proper deposition, characterized by very good reconditioned surface quality, very low oxides, well established metallographic structure, and high resistance to wear and tear [6].

In order to improve the results obtained in the welding reconditioning processes, it is possible that the technological processes of reconditioning are carried out under the conditions of an ultrasonic activation [7].

Currently, two techniques are most common for vibration-assisted machining due to their feasibility and industrial expectations: the Modulation-Assisted Machining and the Ultrasonic Assisted Machining. Modulation-Assisted Machining superimposes a controlled low-frequency oscillation to the cutting process, typically up to 1000 Hz with amplitudes up to 500 µm. 

The mechanics of the chip formation is changed in that way, and cutting is divided into discrete events by means of control of the modulation parameters. Thus, it improves efficiency and process capability in precision machining processes. On the other hand, Ultrasonic Assisted Machining consists in adding high frequency excitation, which can reach 40 kHz, and low vibration amplitude, ranging between 3 and 30 µm [8].

The efficiency of the ultrasonic reconditioning process depends primarily on how ultrasonic energy is introduced into the welding bath. Research has shown that the propagation of ultrasound in the liquid metal bath has considerable influences on the process of transferring the material through the electric arc and on the crystallization process. All these influences are attributed to basic phenomena due to the propagation of ultrasounds in liquid media, namely ultra acoustic cavitation and accelerating the diffusion process [9,10,11]. It is also very important to analyze the longitudinal vibration theory for a bar with a variable transverse section, so as to obtain a greater amplification of the efficiency of ultrasonic vibrations [12].

## 2. Materials and Methods

The base material of the tools used for the refining rubber wastes is a 50WCrV8 alloy steel having the composition shown in Table 1 and the mechanical properties in Table 2.

The reconditioning was carried out by the successive layering of material deposits in order to ensure microstructure regeneration, since the introduction of a new layer of material over the previously deposited layer is obtained by the heating of the latter over the AC3 transformation point and under these conditions a metallographic structure is obtained [13]. It was also taken into account that the reconditioning capacities to be performed by pressing the valves at a rate of 0.5 rpm and the welding head moved at a feed rate of 2.5 mm/min.

The deposition of welded material was achieved by applying the MAG welding process using a type of welding wire G4 Si 1, according to [14] with diameter Ø 1.2 mm as a filler material, the characteristics of the filler material are presented in Table 3 and Table 4.

In order to determine the optimal welding process for the parts used in machine construction, a procedure and set of experiments were established to allow a reliable analysis of the mechanical properties of the welded joints. The MAG welding process is based on welding in shielded gas with a consumable electrode and consists of forming the spring between the consumable wire—underwire shape—and the piece. The welding process can be used in semi-mechanized, mechanized, automated or robotic versions with reverse polarity if the welding source has a rigid external feature [15,16,17]. The control of the electric arc is achieved by means of the self-regulating mechanism while maintaining a constant value of the wire feed in the molten bath. The degree of universality is given by the diversity of welded base materials and the welding position. It should be noted that a high storage rate can be obtained, reaching up to about 10 kg/h, depending on the diameter of the wire. Another feature of this process is the intensive use of the filler material, which leads to slats without slag. The process is currently the most widely used welding process in industrial applications due to economic advantages compared to other methods. 

In order to achieve a high efficiency of the welding reconditioning process and high penetration, the welding parameters presented in Table 5 were used.

**Figure 1:**
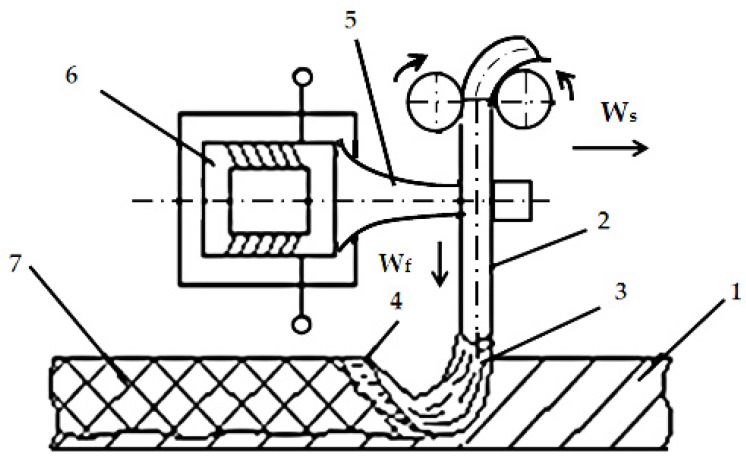
Activation scheme of the welding reconditioning process: 1—basic metal; 2—electrode; 3—electrical arc; 4—liquid metal bath; 5—ultrasonic energy concentrator; 6—ultrasonic transducer; 7—welded seam [18].

The technological process of depositing welding material can be activated ultrasonically, and the presence of ultrasonic energy has multiple advantages due, among others, to the cavitation phenomenon that occurs in the welding bath, and this causes an increase in the cooling rate influencing the solidification process of the materials. The most challenging issue that has been solved in the research was related to the way ultrasonic energy is introduced into the liquid metal bath. Thus, given the considerable size of the tools used in the regenerated rubber refining process, the ultrasonic activation of the electrode used in the welding reconditioning process was considered, as seen in Figure 1 [18].

The effect of ultrasound on the welding reconditioning process is complex and so, in order to highlight the influence of ultrasound on the process of reconditioning in the ultrasonic field, it is necessary to consider the following: the thermal effect, the “softening acoustic” effect and the “hardening acoustic”. Thus, in the ultrasonic welding reconditioning process, a particular importance for the partner materials is the thermal conductivity and the duration of the ultrasonic activation of the deposition process, hence the thermal effect of the ultrasonic field.

By definition, thermal conductivity λ results from the relationship:

(1) $$Q=-\lambda \frac{dT}{dx}dt$$

where: *Q* is the heat flow or the amount of energy crossing a surface unit; *dx*—the temperature gradient along the *x*-direction of the flow; *T*—temperature; *t*—time. The minus sign indicates that the heat flow is from the high-temperature zone to the low-temperature area.

In a metallic material, the thermal flux is transported by the ionic network through its vibrations (thermal conductivity), electron gas (electronic thermal conductivity) and vibration of the grain and phase separation limits (superconducting thermal conductivity). Therefore, it can be said that in the case of ultrasonic activation, the total thermal conductivity *λ* of material is a sum of the form:

(2) $$\lambda =\lambda_{e}+\lambda_{i}+\lambda_{l}$$

where: *λ_e_*—conduction due to thermal agitation of electrons; *λ_i_*—conductivity due to the thermal agitation of the ions; *λ_l_*—the conductivity due to the thermal agitation of grain boundaries and sublimation caused by the propagation of ultrasonic waves.

The differential equation of conductivity through a volume of material is expressed as:

(3) $$\rho c\frac{\partial \theta }{\partial t}=\frac{\partial }{\partial x}\left(\lambda_{x}\frac{\partial \theta }{\partial x}\right)+\frac{\partial }{\partial y}\left(\lambda_{y}\frac{\partial \theta }{\partial y}\right)+\frac{\partial }{\partial z}\left(\lambda_{z}\frac{\partial \theta }{\partial z}\right)$$

where: *θ*—temperature; *λ*—thermal conductivity; *c*—specific heat; *ρ*—density, *t*—time; *x*, *y*, *z*—independent variables that determine the position of a point M, of the material volume in which the temperature is *θ*.

For applications specific to deposition processes by welding, the hypothesis of constant thermophase coefficients is assumed. Thus *λ* and the product *ρc* are chosen for the temperature 1550–1600 °C and the coefficient of convection *α* for the range 1300–1500 °C, the most common cases in practice and thus the Equation (3) becomes:

(4) $$\frac{\partial \theta }{\partial t}=a\cdot \Delta \theta =\frac{\lambda }{\rho c}\cdot \frac{\partial }{\partial x}\left(\frac{\partial^{2}\theta }{\partial x^{2}}+\frac{\partial^{2}\theta }{\partial y^{2}}+\frac{\partial^{2}\theta }{\partial z^{2}}\right)$$

where: *a* represents the temperature transmitting coefficient.

The limit and initial conditions refer to the temperature distribution in the welding pool and heat exchange at the welding pool boundary.

The initial temperature distribution (at *t* = 0) is expressed by:

(5) $$\theta \left(x,y,z,0\right)=\theta_{0}\left(x,y,z\right)$$

which, in the situation *θ*_0_ = 0, indicates that the heat transfer process may be due to internal or external heat sources.

The following conditions are acceptable in practice:

- the surface temperature of the body *θ*_s_ is of the form:
  *θ_s_* = *θ_s_*(*x*, *y*, *z*, *t*) (6)

which, in the case of the isothermal surface becomes *θ_s_* = constant;

- the thermal flow, through the lateral surface of the body *q_s_* is of the form:
  (7) $$q_{s}=q_{s}\left(x,y,z,t\right)=\mathrm{constant}\;\mathrm{or}\;{\frac{\partial \theta }{\partial n}|}_{S}=\mathrm{constant}$$

A particular case is an adiabatic wall when equality occurs:

(8) $$q_{s}=0{\;\mathrm{or}\;\frac{\partial \theta }{\partial n}|}_{S}=0$$

- the heat flux through the lateral surface of the body is expressed with the formula of Newton:
  (9) $$q_{s}=\alpha \left(\theta_{s}-\theta_{0}\right)$$

where: *θ*_0_ is the outside temperature; *α*—coefficient of convection.

Adopting simultaneously:

(10) $$q_{s}=-\lambda {\frac{\partial \theta }{\partial n}|}_{S}$$

from relations (8) and (9) results:

(11) $$\alpha \left(\theta_{s}-\theta_{0}^{}\right)=-\lambda {\frac{\partial \theta }{\partial n}|}_{S}$$

In the conductivity study, analytical methods and numerical methods are used. In the category of analytical methods are included: Fourier method, caloric sources method, and operator method. Determination of temperature for thermal phenomena specific to welding reconditioning processes is applied with good results to the source method.

At the time *t* = 0, the elemental volume *dxdydz* belonging to a volume of material at a temperature *θ*_0_ = 0 is concentrated heat *Q*. An Oxyz Cartesian marker originated in the center of the caloric source, is chosen. The heat transfer process *θ*(*x*, *y*, *z*, *t*) from the concentrated source *Q* is expressed by the equation:

(12) $$\theta \left(x,y,z,t\right)=\frac{Q_{t}}{\rho c{\left(4\pi at\right)}^{3/2}}e^{-\frac{x^{2}+y^{2}+z^{2}}{4at}}$$

where: $a=\frac{\lambda }{\rho c}$; *Q_t_* is the total amount of heat concentrated and equal to:

*Q_t_* = *Q* + *Q_u_* (13)

where: *Q_u_* is the amount of heat introduced by ultrasound probing.

The amount of heat *Q_u_* determined by the presence of ultrasounds contributes to an additional heating of the welding wire. The temperature of the welding wire depends on the intensity of the applied ultrasonic waves and on the activation time, as it has been demonstrated the direct connection between the levels of the ultrasound energy introduced into the welding wire and the thermal energy resulting from its action. The preferential absorption of ultrasonic energy in certain areas enables the resulting thermal energy to be located. In these conditions, the amount of heat *Q_u_*, determined by the presence of ultrasounds, can be calculated depending on the properties of the filler material (metallic wire), namely the mass and the specific heat, as well as the temperature variation determined by the presence of ultrasounds:

*Q_u_* = *m*·*c*·Δ*θ* (14)

where: *m* is the mass of the filler material, *c*—the specific heat of the filler material, Δ*θ*—variation of temperature caused by the presence of ultrasounds

As for the temperature variation Δ*θ*, it can be expressed by a relation of the form:

Δ*θ* = *K*·*A*·*E*·cos(*K*·*x* − *ω*·*t*) (15)

where: *K* = *ω*/*c* = 2 *π*/*λ* is the wave number; *E*—the acoustic energy density; *A*—the amplitude of the acoustic waves; *x*—a coefficient determined by the electrons′ vibrations of the welding wire material; *c*—the propagation velocity of the ultrasonic wave, *λ* the wavelength; ω—angular frequency, *t*—time of activation with ultrasonic energy.

Relation (13) is a solution of the differential equation of conductivity given by relation (1). As the *Q_t_* heat is transmitted through the bath, the temperature at different point′s changes, but the amount of heat remains the same, equal to *Q_t_*.

The acoustic softening effect influences the welding reconditioning process due to the fact that the presence of acoustic energy causes a reduction of the static voltage necessary for the melting of the metal with the increase of the acoustic energy density. Ultrasounds have the same effect on metals as thermal energy, but from a quantitative point of view there is an important difference; while for acoustic energy density densities of 10^15^ eV/cm^3^ can achieve the static strain of metallic deformation at values close to zero, obtaining the same effect by heating requires a thermal energy of 10^22^ eV/cm^3^. Therefore, due to the acoustic softening effect, optimum conditions are created for the creation of a more homogeneous welding bath, the action of thermal energy on the plasticity of metals is less efficient than the action of acoustic energy.

The effect of “acoustic hardening” is the modification of structural properties of materials. Thus, the acoustic hardening appears as a residual effect, after activating the vibration at high intensities specific to the nature of the activated material. As a result of the acoustic hardening, an increase in the displacement density is produced in the metallic material so that, after the end of the ultrasonic activation in the metal, a specific dislocation structure is fixed.

## 3. Results and Discussion

**Figure 2:**
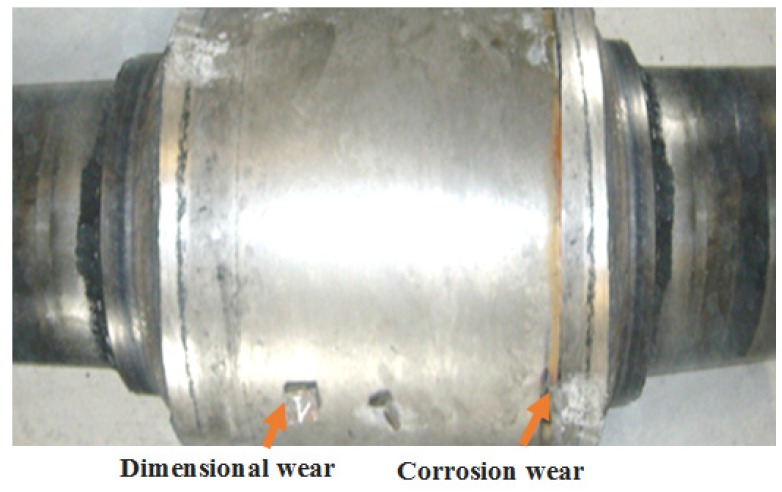
The shape of the tool used in the refining process of the regenerated rubber.

MIG (Metal Inert Gas) or MAG (Metal Active Gas) welding equipment—M-Pro series with removable feed device was used to achieve welding conditioners. The current source used for automatic under-flow welding was a remote-controlled three-phase welding power source built to deliver a high MIG-MAG process, and the MAG process was used in the experiments. Also, this source was used in combination with the control panel realized by ESAB corporation, the A2–A6 Process Controller (PEH). The shape of the tool undergoing the reconditioning process, as well as the types of wear that occur in its case, are shown in Figure 2.

In order to highlight the advantage of using ultrasonic activation in reconditioning and to establish the optimal process of welding reconditioning, both ultrasonic welding reconditioning and reconditioning with ultrasonic activation were analyzed. Thus, the reconditioning of the tool was made by dividing it into four distinct zones, and for each area, the refurbishment was applied taking into account distinct welding regimes. Under these conditions, the refurbishment was done considering the use of four welding regimes, namely:

- reconditioning without ultrasonic activation—zone 1;
- ultrasonic activation reconditioning with the activation frequency f = 18 kHz and amplitude *A* = 30 μm—zone 2;
- reconditioning by ultrasonic activation with the activation frequency f = 20 kHz and amplitude *A* = 45 μm—zone 3;
- reconditioning by ultrasonic activation with the activation frequency f = 22 kHz and amplitude *A* = 60 μm—zone 4.

**Figure 3:**
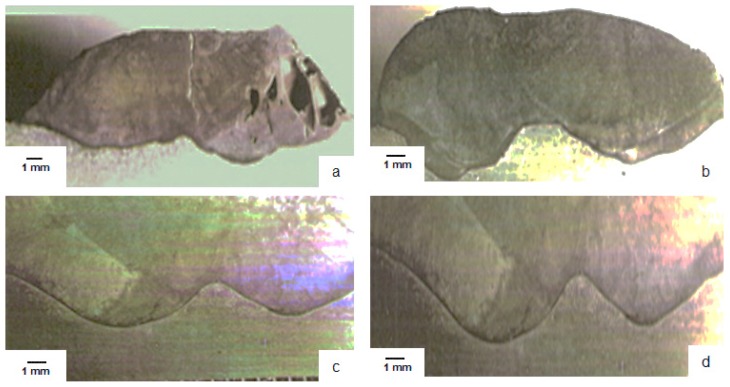
Layers of material deposed in the reconditioning process: (**a**) reconditioning without ultrasonic activation—zone 1; (**b**) reconditioning with ultrasonic activation—activation frequency f = 18 kHz, amplitude *A* = 30 μm—zone 2; (**c**) reconditioning with ultrasonic activation—activation frequency f = 20 kHz, amplitude *A* = 45 μm—zone 3; (**d**) reconditioning with ultrasonic activation—activation frequency f = 22 kHz, amplitude *A* = 60 μm—zone 4.

The layers of welded material were analyzed by the local sectioning of the reconditioned tool, and the shape of the deposited material layers for the 4 welding modes are shown in Figure 3.

The adhesion of the deposited layer over the base material is essential with regard to the mechanical characteristics of the functional surface of the reconditioned part. Analysis of the crystalline structure was done on the metallographic optical microscope for Vision DX51 (Engeneering LTD, Emmering, Germany).

As seen in Figure 3, a much better adhesion of the base material is obtained, and the deposited material layer is obtained when the deposition is done in the reconditioning by welding activated ultrasonic (Figure 3b–d). Also, the application of ultrasonic energy to welding load causes uniform deposition of hard carbons at the boundary between crystalline grains and the elimination of possible oxides, a phenomenon explained by the action of ultrasounds, the preferential absorption of ultrasonic energy at the grain boundary and especially the occurrence of ultra-acoustic cavitation.

Also, due to the research performed, there was a clear influence of the ultrasonic activation on the thermal field and on the crystallization process. Thus, it has been observed that the temperature variation depends on the frequency of the ultrasonic waves, the oscillation velocity of the particle velocity, the acoustic intensity, the duration of activation of the crystallization process and the acoustic impedance (the acoustic impedance is given by the product between the hardness of the material or the liquid alloy and the velocity propagation of ultrasonic waves).

To analyze the thermal field and how it is influenced during ultrasonic activation, an infrared thermography analysis was performed.

**Figure 4:**
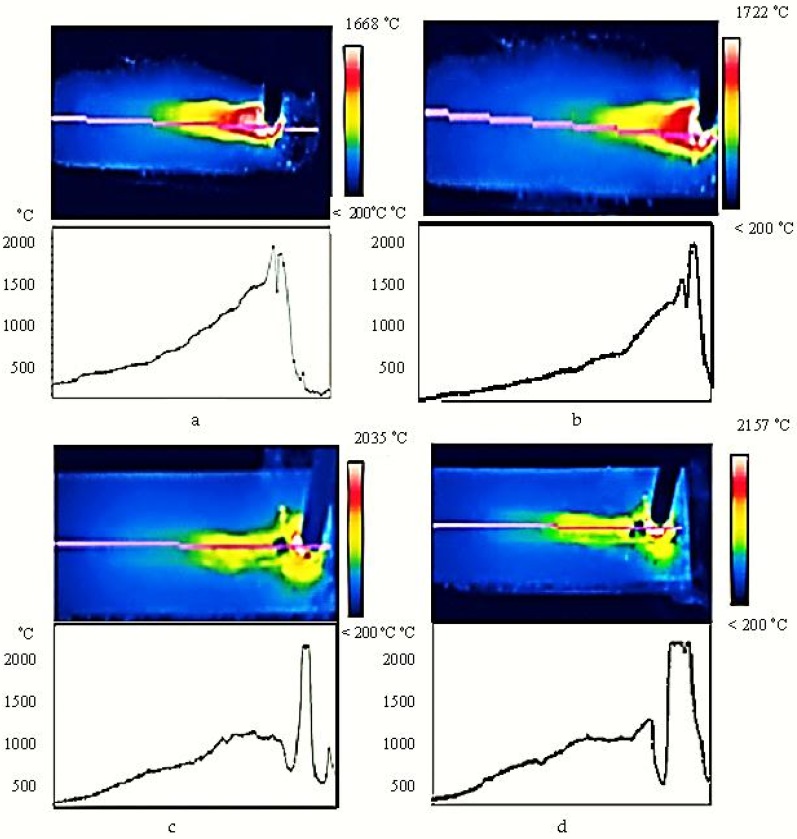
The thermal field and the thermography for the partner materials base material—filler material: (**a**) reconditioning without ultrasonic activation—zone 1; (**b**) reconditioning with ultrasonic activation—activation frequency f = 18 kHz, amplitude *A* = 30 μm—zone 2; (**c**) reconditioning with ultrasonic activation—activation frequency f = 20 kHz, amplitude *A* = 45 μm—zone 3; (**d**) reconditioning with activation of the radio frequency—activation frequency f = 22 kHz, amplitude *A* = 60 μm—zone 4.

Experiments were performed using a THERMACAM SC640 (Electro-Optics Technology, Boston, MA, USA) thermography installation. The experimentation distinguished the thermal field and the thermograph on the couple of material, the base materials—the addition material for welding without ultrasonic activation and with ultrasonic activation, Figure 4.

The treatment of the ultrasonic welding tube leads to the change of the thermal field character, as follows: the ultrasonic current causes intense mixing of metal or liquid alloy rapidly stabilizes the ambient temperature and intensifies the convection diffusion. During fluid temperature uniformity, the heat exchange increases with the base layer and the surrounding environment, which increases the cooling rate.

Under these conditions, it was found that as the frequency increases, the thermal field becomes uniform in a shorter time, the temperature drops more rapidly by accelerating the cooling process at the solid phase-liquid phase interface. Also, as the amplitude increases, the thermal field becomes uniform faster, which is explained by the symmetrical alternating voltages introduced into the welding pool and the thermal hysteresis created by the ultrasonic wave propagation. Experiments have shown that as the activation time increases, a relaxation of the thermal process takes place, so that it is obtained a crystalline structure almost lacking internal thermal tensions, without the need for thermal annealing treatment of strain relief.

The faster uniformization of the thermal field in the case of reconditioning by ultrasonic activation leads to a considerable reduction of the size of the thermal-influenced area. Thus, in the case of welding without the use of ultrasonic activation (Figure 4a), it is observed that there is a thermal field with temperature which is much higher compared to the situation where ultrasonic welding reconditioning is used. The smallest high temperature zone is obtained in the case of reconditioning with ultrasonic activation with the activation frequency f = 22 kHz and the amplitude *A* = 60 μm, Figure 4d, and thus the thermally influenced area has the smallest width in this reconditioning variant.

The analysis of the thermal field (Figure 4) allows a conclusion to be drawn that the ultrasonic activation has a very important influence on the welding thermal field and on the process of crystallization of the deposited material and implicitly on its porosity. Thus, it has been inferred that ultrasonic activation causes a temperature variation in the materials (deposited material and base material) and, consequently, influences the porosity in the layer of deposited material. Obtaining a reduced porosity in the layer of deposited material can be achieved if the optimum frequency values are adjusted which is activated by the welding electrode (f) and the particle velocity amplitude (*A*).

From the analysis of the thermal field and the thermograph in the partner materials base material—material of addition (Figure 4), we can see that the slowest uniformization of the thermal field was obtained in the case of reconditioning without ultrasonic activation (Figure 4a) the rapid equalization of the thermal field was obtained in the case of reconditioning with ultrasonic activation with the activation frequency f = 22 kHz and amplitude *A* = 60 μm, Figure 4d.

As a result of the research, it was found that as the particle oscillation amplitude increased, a faster thermal uniformization was obtained, which is explained by the symmetrical alternating voltages introduced into the welding pool and the thermal hysteresis created by the ultrasonic wave propagation, and this influences the way the adhesion between the base material and the adduct material and the porosity of the adduct material is produced. Thus, it was observed that the lowest porosity of filler material was obtained if the particle oscillation amplitude was *A* = 60 μm (Figure 4d).

**Figure 5:**
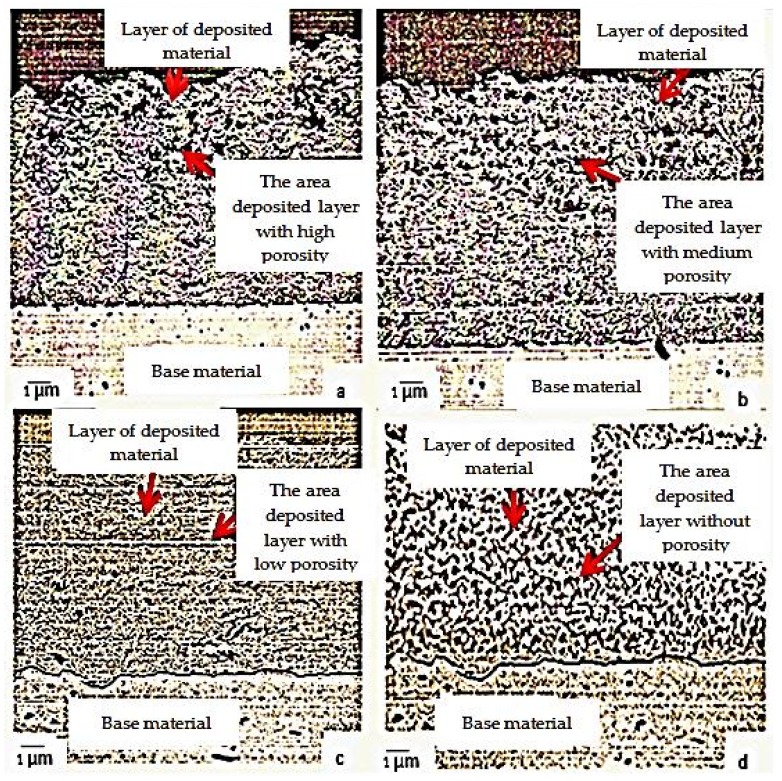
Porosity in the layer of material deposited under the following conditions: (**a**) reconditioning without ultrasonic activation; (**b**) reconditioning with ultrasonic activation with the activation frequency f = 18 kHz and amplitude *A* = 30 µm; (**c**) reconditioning with ultrasonic activation with the activation frequency f = 20 kHz and amplitude *A* = 45 µm; (**d**) reconditioning with ultrasonic activation with activation frequency f = 22 kHz; amplitude *A* = 60 µm.

Under these conditions, the experimental research has shown that the presence of ultrasounds also causes a relaxation of the thermal process, and thus a crystalline structure is obtained that does not have internal thermal stresses or they have a very low value. Thus, conditions are created to avoid the application of a thermal annealing treatment, as demonstrated by the analysis of the metallographic structure of the samples obtained in the case of reconditioning by ultrasonic field welding (Figure 5). This is very important for these types of tools because they are very large and the need to apply heat treatment after the welding load would lead to a considerable increase in energy consumption and a decrease in productivity.

Also, obtaining a low porosity in the adhesion material of a good adhesion between the deposited material and the filler material may also be influenced by the acoustic intensity and acoustic impedance.

The analysis of the thermal field showed that the maximum temperature occurs at reconditioning by welding (Figure 4d), and the highest temperature was obtained if the ultrasonic waves had the highest frequency and the highest amplitude. This is a confirmation of Equation (13), which shows that ultrasounds increase heat in the welding process and, implicitly, temperature. Also, the form of Equation (15) is confirmed by the fact that the maximum welding temperature was obtained if the welding reconditioning process was activated ultrasonically with an activation frequency f = 22 kHz and an amplitude of *A* = 60 μm.

The research also investigated the effects of ultrasonic propagation in the welding pool on the metallographic structure of the filler material layer and of the assembly obtained under different deposition conditions of the layer of the filler material. Thus, samples were taken from the four distinct areas that were reconditioned by welding, each of which was analyzed metallographically, taking into account especially the porosity of the layer of deposited material, and also the metallographic constituents obtained in it (Figure 5).

Porosity in the deposited material layer is very important from the point of view of how the tool to be reconditioned will behave in operation. Thus, in the case of a large porosity, various corrosive agents will enter the surface layer of the tool which will further determine the occurrence of the phenomenon of cracking corrosion. Under these conditions, by analyzing the metallographic structure, we could observe the porosity distribution in the layer of deposited material. Figure 5 shows the porosity that occurs during reconditioning by welding. Thus, it was noticed that if reconditioning by welding is done without the use of ultrasounds (Figure 5a) the porosity is much higher than the variant where the reconditioning is done by ultrasonic welding (Figure 5b–d). From the analysis of the metallographic structure of the layers of deposited materials, it was ascertained that, as the frequency increases, the thermal field becomes uniform in a shorter time, the temperature decreases more rapidly by accelerating the cooling process at the liquid phase interface solid phase, this resulting in a better homogeneity of the material, and for an activation frequency f = 22 kHz and an amplitude of particle oscillation *A* = 60 μm, the best homogeneity of the deposited material was obtained without the presence of pores therein (Figure 5d).

Also, achieving a lower porosity in the deposited material layer is very important because a high porosity can cause an exfoliation of the layer of material deposited through the reconditioning. Thus, it was necessary to find an optimal technical solution for the deposition of the filler material and, from the researches carried out, it was found that a much lower porosity is obtained in the filler material in case of reconditioning by ultrasonic welding, and this can be explained especially through the thermal effect of ultrasounds when used in reconditioning by welding.

Activating the welding process to reconditioning the tools used in the regenerated rubber production process allows for better intermetallic bonding to a larger diffusion, to avoid defects in the transition zone between the base material and the deposited material layer and better functional and technological characteristics of these types of tools. Thus, in the experimental research it is found that as the frequency and amplitude of the ultrasonic waves increase, so does the diffusion process increase. Under these conditions there is not the possibility to occurrence defects of the porosity type and the lack of adhesion between the base material and the deposited material, as often occurs in the case of deposition of material layers by welding the presence of reconditioning by welding without activated ultrasonic.

From the analysis of the metallographic structures, Figure 5, there was a substantial change of the granular structure, namely the modification of the grain size and uniformity according to the technological and acoustic parameters. Thus, the finest granulation is obtained by reconditioning in the ultrasonic field compared to the reconditioning without ultrasonic activation. Ultrasonic reconditioning results in the addition of a much finer granulation, but also a primary crystallization structure made up of very fine ledeburite grown in a predominant martensite matrix, and this causes the hardness of the deposited layer to increase, concurrently with better plasticity and better tenacity.

Thus, the hardness of the deposited material layer, the hardness of the Vickers HV10 with the Tukon 1102/1202, was determined within the research. The hardness was measured at three distinct points for each area, and the hardness values measured are shown in Table 6.

From the analysis of the measured hardness values, a substantial increase of the hardness was found in the ultrasonic activation of the welding bath, this being done simultaneously with the fracturing of the structure in the case of the ultrasonic welding load.

It has also been observed that in the melting zone, where dilution of the feed material takes place in the base material, the amount of ledeburite does not decrease abruptly. Depending on how dilution of the feed material takes place in the base material, the amount of ledeburite varies with the increase in dilution.

At the same time, the presence of ultra-acoustic cavitation produces strong shock waves, and thus easily obtains a dispersion of metal, which allows the creation of conditions to obtain mixtures of metals that cannot normally be obtained. Thus, by applying the welding refurbishment, a considerable increase in the lifetime of the tools used in the regeneration of the regenerated rubber is achieved.

## 4. Conclusions

The reconditioning by ultrasonic welding of the parts used in the refining process of the regenerated rubber can be used in very good conditions due to the following advantages:

- the thermal field and the thermograph analysis of the partner materials base material—filler material has shown that the fastest heat uniformization of the thermal field is obtained by reconditioning in the ultrasonic field;
- as the frequency of ultrasonic oscillation increases, the heat field becomes uniform in a shorter time, the temperature decreases more rapidly by accelerating the cooling process at the solid-phase liquid phase interface;
- as the amplitude increases, the thermal field is uniformized faster, which is explained by the symmetrical alternating voltages introduced into the welding pool and the thermal hysteresis created by the ultrasonic wave propagation;
- the absence of ultrasonic waves in conventional welding reconditioning results in a long-term maintenance of the heat in the added material and the base material and this causes the magnitude of the heat-affected zone to be much higher at the conventional reconditioning in relation to welding reconditioning with ultrasonic activation;
- a very good porosity between the base material and the adduct is obtained, and the best results were obtained if the ultrasonic activation was done with an activation frequency f = 22 kHz, the particle vibration amplitude was *A* = 60 μm;
- the material of addition has a homogeneous primary crystallization structure without the presence of pores, which occur very frequently in the case of welding without ultrasonic activation;
- the presence of ultrasound determines the conditions for an optimal dilution of the filler material in the base material, and this allows a substantial increase in the adhesion between the two materials;
- for the elimination of flaws such as porosities in the filler material it is advisable to work with an increased oscillation frequency and increased amplitude;
- the crystalline grains from the deposited material area, and also from the thermal influenced area, have a uniform size, creating the conditions for a homogeneous structure;
- the hardness of the material in the deposited layer increases considerably in the case of reconditioning with ultrasonic activation, and the highest value was obtained for reconditioning with ultrasonic activation: activation frequency f = 20 kHz, amplitude *A* = 60 μm;
- the research proved that the use of ultrasonic waves in the welding reconditioning process creates conditions for obtaining technical and economic results superior to the classic welding reconditioning.

## Figures and Tables

**Table 1:** The chemical composition of 50WCrV8 alloy steel.

C %	Si %	Mn %	P %	S %	Cr %	V %	W %
0.45–0.55	0.7–1.00	0.15–0.45	0.030	0.030	0.90–1.20	0.10–0.20	0.70–1.20

**Table 2:** The mechanical properties of 50WCrV8 alloy steel.

Young′s Modulus MPa	Tensile Strength MPa	Elongation %	Fatigue MPa	Yield Strength MPa
200,000	650–880	8–25	275	350

**Table 3:** The chemical composition of the welding wire G4 Si 1.

C %	Si %	Mn %
0.06–0.14	0.8–1.2	1.6–1.9

**Table 4:** The mechanical properties of the welding wire G4 Si 1.

Yield Strength MPa	Tensile Strength MPa	Impact Resistance (ISO–V/40°)/J	Elongation at Break (Lo = 5Do)/%
min. 500	560–720	min. 47	min. 25

**Table 5:** The parameters of the welding process.

CO_2_ Protective Gas %	Type of Transfer	Wire Feed Speed, W_f_ m/min	Welding Current Intensity I_W_/A	Welding Voltage U_w_/V	Welding Speed, W_s_ m/min	Protective Gas Flow Rate D_g_ L/min	Linear Energy KJ/mm
100	spray-arc	10	300	30	7	20	0.86

**Table 6:** Vickers HV10 hardness values of the deposited material layer.

Reconditioning Without Ultrasonic Activation	Reconditioning with Ultrasonic Activation: Activation Frequency f = 18 kHz, Amplitude *A* = 30 μm	Reconditioning with Ultrasonic Activation: Activation Frequency f = 20 kHz, Amplitude *A* = 45 μm	Reconditioning with Ultrasonic Activation: Activation Frequency f = 20 kHz, Amplitude *A* = 60 μm
755–750–769	935–932–940	955–952–950	998–995–989
